# Dermatomyosite et panniculite: place des immunoglobulines

**DOI:** 10.11604/pamj.2016.23.262.6213

**Published:** 2016-04-29

**Authors:** Nadia Ben Abdelhafidh, Sana Toujeni, Asma Kefi, Najeh Bousetta, Sameh Sayhi, Imen Gharsallah, Salah Othmani

**Affiliations:** 1 Service de Médecine Interne Hôpital Militaire de Tunis, Montfleury, Tunisie

**Keywords:** Dermatomyositis, panniculitis, immunosuppressants drugs, immunoglobulins, Dermatomyositis, panniculitis, immunosuppressants drugs, immunoglobulins

## Abstract

La panniculite est une maladie inflammatoire du tissu adipeux sous-cutané rarement associée à la dermatomyosite. Elle peut survenir avant, après ou en même temps que l'atteinte musculaire. Dans la plupart des cas, l’évolution de la panniculite et des autres atteintes de la dermatomyosite est favorable sous traitement corticoïde et/ou immunosuppresseur. Nous rapportons le cas d'une patiente âgée de 48 ans ayant présenté des lésions de panniculite précédant de 2 mois les signes musculaires. L'atteinte cutanée était résistante au traitement corticoïde associés aux immunosuppresseurs ce qui a nécessité le recours au traitement par Immunoglobulines polyvalentes permettant ainsi une amélioration à la fois de l'atteinte cutanée et musculaire.

## Abstract (en)

Panniculitis is an inflammatory disease of subcutaneous adipose tissue which is rarely associated with dermatomyositis. It can occur before, after or simultaneously with muscle damage. In most cases, the evolution of panniculitis and of other dermatomyositis affections is favorable with corticosteroids and/or immunosuppressants. We report the case of a 48 year-old patient who developed panniculitis lesions 2 months before having muscular signs. Skin involvement was resistant to corticosteroid treatment associated with immunosuppressants drugs. This led to the use of polyvalent immunoglobulin treatment improving both skin and muscle damage.

## Introduction

La dermatomyosite (DM) est une myopathie inflammatoire regroupant des signes musculaires et cutanés telles que les papules de gottron et l’érythème périorbitaire. La panniculite qui se présente cliniquement sous forme de lésions nodulaires érythémateuses est rarement associée à la DM. Elle peut survenir avant, après ou en même temps que l'atteinte musculaire. Dans la plupart des cas, l’évolution de la panniculite et des autres atteintes de la DM est favorable sous traitement corticoïde et/ou immunosupresseur. Nous rapportons le cas d'une patiente âgée de 48 ans ayant présenté des lésions de panniculite précédant de 2 mois l'apparition des signes musculaires et ayant résisté aux corticoides associés au méthotrexate ce qui a nécessité le recours à un traitement par Immunoglobulines polyvalentes.

## Patient et observation

**Figure 1:**
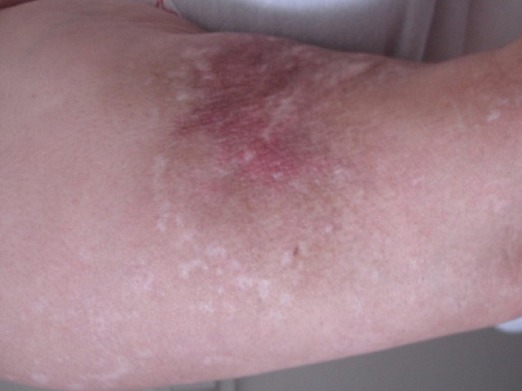
Nodules dermo-hypodermiques érythémateuses au niveau du bras

**Figure 2:**
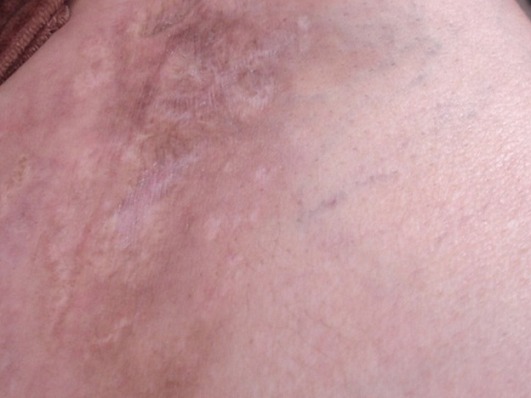
Nodules hyperchromiques de la cuisse

**Figure 3:**
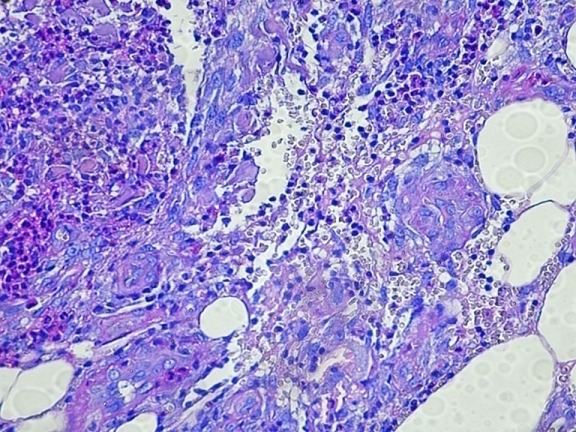
HE x 400: tissu adipeux siège d'une réaction inflammatoire à PN

**Figure 4:**
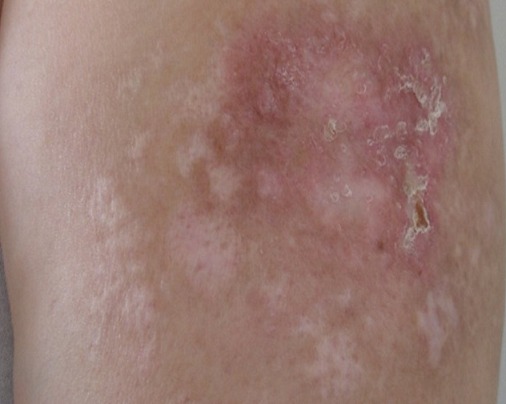
Evolution sous traitement des lésions du bras: disparition des nodules et des desquamations cutanées

Patiente âgée de 47 ans, sans antécédent pathologique notable, hospitalisée pour myalgies avec faiblesse musculaire prédominant aux ceintures pelvienne et scapulaire. L'atteinte musculaire était précédée de plusieurs placards érythémato-nodulaires douloureux siégeant au niveau des 2 bras (Figure 1) et des 2 cuisses (Figure 2) apparus 2 mois auparavant associés à un érythème violacé des paupières et du décolleté et un livédo des membres inférieurs. Le testing musculaire avait conclu à un déficit musculaire proximal et l’électromyogramme à un tracé myogène diffus. Le dosage des enzymes musculaires montrait une créatine phosphokinase (CPK) élevée à 1953 UI/L soit 15 fois la normale et une lactate déshydrogénase à 6 fois le normale. Le bilan immunologique (anticorps anti nucléaires, anti J01, anti Sm, anti RNP, anti SSa et anti SSb) était négatif. La biopsie des lésions nodulaires avait mis en évidence: un épiderme en partie ulcérée, la couche basale intacte ne comportant pas de corps hyalin. Le derme sous jacent comporte une inflammation polymorphe et discrète de siège péri vasculaire. Ces derniers sont de morphologie normale, non ectasiques et non congestifs. L'hypoderme est caractérisé par une discrète inflammation de siège septale et panniculaire. Elle est composée par des petits lymphoplasmocytes réguliers associés à de rares polynucléaires (Figure 3). Par ailleurs absence de cellules lymphoïdes suspectes ou de signes histologiques de malignité avec une immunohistochimie (CD3, CD20, CD4, CD8) négative. Le diagnostic d'une panniculite associée à une DM était retenu. Le bilan clinico-biologique effectué à la recherche d'une néoplasie sous-jacente et comportant: l'examen physique, les marqueurs tumoraux, la fibroscopie digestive, la radiographie du thorax, l’échographie et le scanner thoraco-abdomino-pelvien était négatif. Le bilan lésionnel de la dermatomyosite n'a pas montré d'atteinte viscérale associée: les explorations fonctionnelles respiratoires étaient normales, le scanner thoracique ne montrait que des images d'atélectasies banales et l’échographie cardiaque était sans anomalies. Une corticothérapie à base de boli de méthyl prednisolone (1 g/jour pendant 3 jours) relayés par une corticothérapie orale à la dose de 1 mg/Kg/j et du méthotrexate à la dose de 30 mg/sem étaient prescrits. L’évolution au bout de 4 semaines était marquée par une nette amélioration de l'atteinte musculaire (disparition des myalgies, du déficit musculaire, normalisation des enzymes musculaires) mais résistance et aggravation des lésions de panniculite devenues plus étendues avec plusieurs épisodes de surinfections ayant nécessité des hospitalisations itératives. La dapsone à la dose de 100mg/jour pendant 2 mois et les antipaludéens de synthèse( Hydroxychloroquine 400mg/jour) étaient également inefficaces. Devant la résistance de la panniculite aux différentes thérapeutiques à savoir: corticoïdes, Méthotrexate, Dapsone et Hydroxy chloroquine, un traitement par Immunoglobulines polyvalentes intraveineuses (IgIV) était introduit (6 cures à la dose de 2g/Kg/cure). L’évolution était spectaculaire dès la première cure avec stabilisation puis nette régression des lésions de panniculite (Figure 4). Trois mois après l'arrêt des Ig IV, il y a eu une reprise évolutive des lésions de panniculite nécessitant la réintroduction mensuelle d'Ig IV (6 cures) avec de nouveau une rémission dès la première cure.

## Discussion

La dermatomyosite est une connectivite associant une atteinte musculaire et des manifestations cutanées dont certaines sont spécifiques. La panniculite, maladie inflammatoire du tissu adipeux sous-cutané, est rarement observée au cours de la DM. Le premier cas d'association dermatomyosite-panniculite a été décrit en 1924 par Weber et Gray et depuis, seulement une vingtaine de cas sporadiques ont été rapportés depuis [1–3]. Une prédominance féminine est retrouvée dans 75% des cas. L’étiopathogénie de la panniculite au cours de la DM n'est pas encore bien élucidée [1, 4]. La dermatomyosite peut entrainer des remaniements du tissu graisseux pouvant évoluer vers une panniculite lobulaire [4]. Cette hypothèse est suggérée par la constatation de remaniements microscopiques du tissu graisseux sous cutané observés au cours des biopsies cutanées et musculaires chez les patients atteints de DM. La panniculite se présente cliniquement sous forme de lésions nodulaires érythémateuses et douloureuses siégeant essentiellement au niveau des bras, des cuisses, des fesses et de l'abdomen pouvant évoluer vers la lipoatrophie et la calcification [2, 5]. Elle peut apparaître en même temps que les autres manifestations de la DM, la précéder comme c'est le cas de notre patiente ou la suivre. La panniculite peut survenir 14 mois avant le diagnostic de la DM ou 5 ans après cette dernière [1], d'où l'intérêt d'un suivi régulier et du dosage des enzymes musculaires chez les patients atteints de panniculite. Le diagnostic est basé sur l'histologie qui montre typiquement une panniculite lobulaire avec infiltration lymphocytaire associée à une nécrose graisseuse. Des remaniements fibrosants ou des lésions de vascularite peuvent être associés [6]. Sur les 20 cas rapportés dans la littérature, 3 avaient une néoplasie associée. Il s'agissait d'un rhabdomyosarcome [7], d'un carcinome lympho-épithélial like [8] et d'un adénocarcinome ovarien dans un dernier cas [1]. Le traitement de la panniculite associée à la DM n'est pas bien codifié. La majorité des auteurs recommandent une corticothérapie de première ligne à base de boli de méthylprednisolone à la dose de 15 mg/kg/j pendant 3 jours relayés par une corticothérapie orale. L'administration orale des corticoïdes peut être indiquée d'emblée à la dose de à 0,5 à 1 mg/kg/j avec une dégression lente. Les antipaludéens de synthèse tels que l'hydroxychloroquine n'ont pas montré leur efficacité dans ce cadre contrairement à la panniculite lupique [5]. Les agents immunosuppresseurs et surtout le méthotrexate ou la ciclosporine A peuvent être utilisés soit d'emblée ou en 2 ^ème^ intention en cas d’échec de la corticothérapie seule [3, 4]. Dans la littérature 4 cas d'association panniculite et DM étaient traités favorablement par une association corticoïdes et méthotrexate [1]. Une étude récente de Hansen et al [5] a considéré l'adjonction de la corticothérapie à un agent immunosuppresseur d’épargne comme le traitement de première ligne de l'association panniculite DM. Les immunoglobulines intraveineuses ont montré leur efficacité dans l'atteinte musulaire et cutanée de la DM en dehors de la panniculite. Des cures mensuelles à la dose de 2mg/kg/cure sont généralement recommandées. Un seul cas de panniculite associée à la DM rapporté par Sabroe et al [9] a répondu favorablement aux Ig IV. La dose prescrite était 2 mg/kg/cure en association avec la cyclosporine A et la corticothérapie [5]. L’évolution de la panniculite dans les cas publiés est très souvent parallèle à celle de la dermatomyosite quelque soit la thérapeutique utilisée [2, 4, 6]. Le Tableau 1 résume les caractéristiques thérapeutiques et évolutives des panniculites associées aux dermatomyosites dans la littérature [1–4, 6, 9, 10]. La particularité de notre observation tient au fait que la panniculite a continué à évoluer pour son propre compte alors que les autres manifestations de la DM avaient totalement régressé sous corticoïdes et méthotrexate.

**Tableau 1:** Caractéristiques thérapeutiques et évolutives des panniculites associées aux dermatomyosites dans la littérature

Cas	Réf	Délai d'apparition de la panniculite par rapport à la DM	Traitement	Réponse au traitement	Délai de réponse
23 ans (F)	6	Après	NP	ND	
29 ans (F)	6	Concomitant	NP	ND	
19 ans (H)	10	15 mois après	Prednisone (1 mg/kg/j), AZT (3 mg/kg/j)	Favorable	4 sem
44 ans (F)	3	2,5 mois avant	Prednisone (1 mg/kg/j), MTX (7,5 mg/sem); AZT (100 mg*5/sem); IgIV 25 g/mois	Favorable sous Ig IV	6 mois
42 ans (H)	9	5 ans après	Prednisone, CYC A, HCQ, IgIV (2mg/kg/mois)	Favorable	5 mois
42 ans (F)	4	17 mois après	Prednisone (1 mg/kg/j), MTX (7,5 mg/sem); CYC A (200 mg/j)	Favorable	
80 ans (F)	4	10 mois après	Prednisone (1 mg/kg/j), AZT (2 mg/kg/j)	Favorable	
18 ans (F)	2	2 ans après	Vancomycine, ampicilline, métronidazole	Favorable	6 sem
63 ans (F)	1	25 mois après	Prednisone (20mg/j), MTX	Récidive	
Notre cas		2 mois avant	Prednisone, MP, HCQ, Dapsone, MTX, Ig IV	Favorable	

MP: méthylprednisolone, NP: non précisé, HCQ: hydroxychloroquine, MTX: méthotrexate, Ig IV: immunoglobulines intraveineuses, AZT: Azathioprine, CYC A: cyclosporine A; Sem: semaine.

## Conclusion

Les manifestations dermatologiques de la DM répondent favorablement aux traitements corticoïdes associés ou non aux immunosuppresseurs. La panniculite, rarement associée, est elle aussi sensible à ce type de traitement. Nous rapportons ici une observation particulière avec une résistance à des thérapeutiques usuelles (corticoïdes, IS, APS, Dapsone) et une sensibilité aux Ig IV.
